# Doughnut-shaped endoscopic submucosal dissection for circumferential ileocecal valve adenoma

**DOI:** 10.1055/a-2353-6201

**Published:** 2024-07-15

**Authors:** Yugo Suzuki, Kosuke Nomura, Hanako Inoue, Daisuke Kikuchi, Akira Matsui, Shu Hoteya

**Affiliations:** 1 13600 Department of Gastroenterology, Toranomon Hospital, Tokyo, Japan

Endoscopic submucosal dissection (ESD) has not become established as a standard technique for treatment of ileocecal valve (ICV) lesions, and its efficacy has been reported as limited because of its technical difficulty and the time required 1 2 3 . Regarding circumferential lesions in particular, there have been few reports of treatment with ESD 4 .

A 40-year-old woman with type B cirrhosis and diabetes mellitus underwent screening lower gastrointestinal endoscopy and was found to have a large (30-mm) type 0-IIa lesion extending around the entire circumference of the ICV. The lesion was endoscopically diagnosed as adenoma by narrow-band imaging magnification and chromoendoscopy with crystal violet ( Fig. 1 ). Tissue biopsy confirmed the diagnosis of adenoma, and we performed ESD ( Video 1 ). The ESD procedure was performed using a PCF-H290TI (Olympus, Tokyo, Japan) and DualKnife J (KD-655Q; Olympus). A VIO 300D system (Erbe, Tübingen, Germany) was used as the electrosurgical unit. A multiloop traction device (Boston Scientific, Tokyo, Japan) was used to perform traction from the anorectal side. The lesion was resected en bloc without any adverse events, and histopathology confirmed R0 resection of a large tubular adenoma measuring 30 × 28 mm ( Fig. 2 ).

**Fig. 1:**
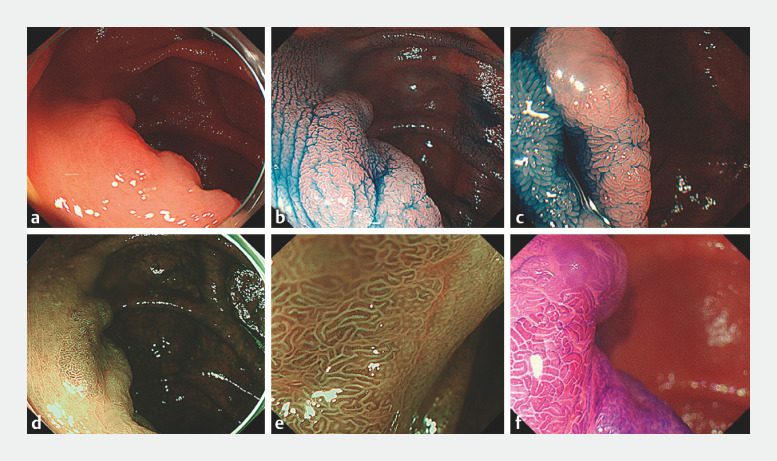
Pretreatment endoscopic evaluation. **a** An erythematous 0-IIa lesion is seen at the ileocecal valve. **b, c** The margin of the 0-IIa lesion is clearly delineated after spraying with 0.4% indigo carmine dye ( **b** anal side; **c** cecal side). **d** Narrow-band imaging (NBI). The lesion appears as a pale brownish area. **e** Magnifying NBI. A regular surface pattern and vessel pattern are observed; the lesion was diagnosed as Japan NBI Expert Team classification type 2A. **f** Magnified chromoendoscopy with crystal violet staining showed a type IV pit pattern.

**Fig. 2:**
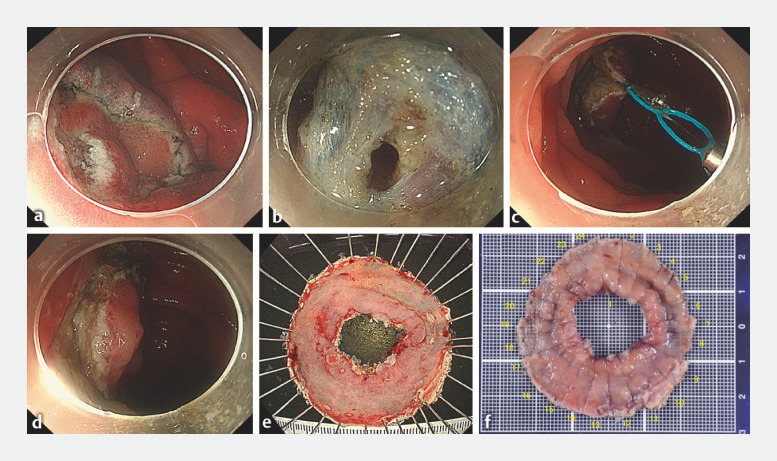
Endoscopic procedure. **a** Circumferential dissection at the ileal border of the lesion. **b** Creation of a tunnel. **c** Attaching the multiloop traction device to the mucosal edge to elevate it. **d** Endoscopic submucosal dissection ulcer after resection. The lesion was resected en bloc without adverse events. **e** Doughnut-shaped endoscopic submucosal dissection specimen. The specimen size was 50 × 50 mm. **f** Pathology showed a tubular adenoma measuring 30 × 28 mm with negative margins.

Successful doughnut-shaped endoscopic submucosal dissection for a circumferential adenoma located at the ileocecal valve.Video 1

Complete resection rates for endoscopic treatment of superficial neoplasms extending into the ileocecal valve are low, and tumor recurrence is consequently a problem 1 . Curative resection by ESD, as in this case, has the major advantages of being less invasive and preserving function. No case of stenosis has been reported for total-circumferential lesions of the ICV among the 9 cases treated with ESD reported to date, including this case 4 5 . The ICV is where the ileal and cecal lumens meet vertically; therefore, the contraction tension during ulcer healing after ESD may radiate outward, which may help stretch the ICV open, without development of stricture 4 .

In conclusion, the doughnut-shaped ESD appears to be a safe, feasible, and effective method for removing circumferential lesions of the ICV.

Endoscopy_UCTN_Code_TTT_1AO_2AG_3AD

## Notes

Endoscopy E-Videos https://eref.thieme.de/e-videos
*E-Videos* is an open access online section of the journal *Endoscopy* , reporting on interesting cases and new techniques in gastroenterological endoscopy. All papers include a high-quality video and are published with a Creative Commons CC-BY license. Endoscopy E-Videos qualify for HINARI discounts and waivers and eligibility is automatically checked during the submission process. We grant 100% waivers to articles whose corresponding authors are based in Group A countries and 50% waivers to those who are based in Group B countries as classified by Research4Life (see: https://www.research4life.org/access/eligibility/ ). This section has its own submission website at https://mc.manuscriptcentral.com/e-videos .
